# The Mixed Finite Element Multigrid Method for Stokes Equations

**DOI:** 10.1155/2015/460421

**Published:** 2015-04-07

**Authors:** K. Muzhinji, S. Shateyi, S. S. Motsa

**Affiliations:** ^1^Department of Mathematics, University of Venda, Private Bag X5050, Thohoyandou 0950, South Africa; ^2^Department of Mathematics, University of KwaZulu-Natal, Private Bag X01, Scottsville, Pietermaritzburg 3209, South Africa

## Abstract

The stable finite element discretization of the Stokes problem produces a symmetric indefinite system of linear algebraic equations. A variety of iterative solvers have been proposed for such systems in an attempt to construct efficient, fast, and robust solution techniques. This paper investigates one of such iterative solvers, the geometric multigrid solver, to find the approximate solution of the indefinite systems. The main ingredient of the multigrid method is the choice of an appropriate smoothing strategy. This study considers the application of different smoothers and compares their effects in the overall performance of the multigrid solver. We study the multigrid method with the following smoothers: distributed Gauss Seidel, inexact Uzawa, preconditioned MINRES, and Braess-Sarazin type smoothers. A comparative study of the smoothers shows that the Braess-Sarazin smoothers enhance good performance of the multigrid method. We study the problem in a two-dimensional domain using stable Hood-Taylor *Q*_2_-*Q*_1_ pair of finite rectangular elements. We also give the main theoretical convergence results. We present the numerical results to demonstrate the efficiency and robustness of the multigrid method and confirm the theoretical results.

## 1. Introduction

This study considers the numerical solution of the large scale linear algebraic system arising from the discretization of the partial differential equations. The discretization is achieved by the finite element method. For positive definite linear systems, linked to the Poisson equations, the multigrid (MGM) methods are proven to be the most effective and fast methods [1, 2]. However it is more challenging for linear indefinite algebraic systems. In this paper we consider multigrid methods for solving linear indefinite algebraic system of equations arising from the mixed finite element discretization of the steady state Stokes problem:

(1) $$\begin{array}{c} -\Delta u+\nabla p=f,\;\text{in}\;\Omega , \end{array}$$

(2) $$\begin{array}{c} \mathrm{div}u=0,\;\text{in}\;\Omega , \end{array}$$

(3) $$\begin{array}{c} u=0,\;\mathrm{on}\;\partial \Omega , \end{array}$$

where **u** is a velocity field, *p* represents pressure, and **f** is an external force field. The problem is considered with (1)–(3) defined on the domain *Ω*⊆*ℛ* ^2^ with boundary ∂*Ω*.

The main goal of this work is to construct and analyze numerical methods that produce an appropriate solution to the Stokes problem. The main thrust is to apply an iterative method, the multigrid method, to solve the linear system of equations that arise from the discretization of the Stokes equations. The MFEM applied to (1)–(3) with carefully chosen finite element spaces results in the algebraic system which must be solved. The velocity variable **u** together with the pressure variable *p* is the solutions of the system. We discretize the domain of the Stokes problem by the rectangular grids with a pair of conforming mixed finite element spaces that are inf-sup stable. In our experiment we use Hood-Taylor *Q*_2_-*Q*_1_ pair as used by [3]. The process produces a symmetric indefinite system of linear algebraic equations. In this paper we study an efficient solver for this system. This work on multigrid method has been motivated by the need to effectively and efficiently solve large application problems. The multigrid method has been shown to be very efficient and successful in solving control problems [1, 2, 4–6] and elliptic partial differential equations [7–9] in an accurate and computationally efficient way. The multigrid method has been applied to problems discretized by the finite difference method and widely by finite element method [3, 8, 10–14]. The effectiveness of the multigrid method depends on the correct choice of the smoothers. Various smoothers have been suggested in literature weighted Jacobi, Gauss Seidel [11], Ilu [8], Vanka-type [9, 12, 13, 15], Braess-Sarazin-type ([13, 16–18]), Semi implicit method for pressure linked equations [15], SOR/Richardson [18], and inexact Uzawa [18]. It is the purpose of this study to apply the multigrid solver to the Stokes problem with the following iterative solvers as smoothers: Braess-Sarazin, inexact Uzawa, preconditioned MINRES, and the distributed Gauss Seidel. The inner solver of these smoothers can also be taken as the multigrid method for the definite subsystems. There is no work known where a comparative study is made on the effects of these four smoothers on the performance of the multigrid method for indefinite systems. The first step is to transform the continuous problem to the discrete system and apply the MFEM that produces the linear algebraic system on which the multigrid method is developed, analyzed, and finally numerically and computationally implemented.

The key features and ingredients of the multigrid method are smoothing and coarse grid correction that involves the intergrid transfers and a solution correction step. The main results of the work are the convergence of the multigrid method in calculating the velocity and pressure variables in an appropriate norm which is based on the smoothing and approximation properties [9, 18]. The rest of the paper is organized as follows. In Section 2 we give the discrete system of the Stokes problem by mixed finite element method. In Section 3 the iterative solution technique, the geometric method, and smoothers are outlined. The known theoretical convergence analysis results are also outlined. In Section 4 a numerical experimental and comparative analysis on the effects of smoothers on the performance multigrid method is presented and discussed and the conclusion is given.

## 2. The Stokes Discrete System

For the discretization of the Stokes equations in the domain *Ω* we need to transform the system (1)–(3) to the weak variational form. For the weak variational formulation of the Stokes equations we define the following solution and test spaces:

(4) $$\begin{array}{c} H^{1}\left(\Omega \right):=\left\{u:\Omega ⟶R\;|\;u,\nabla u\in L^{2}\left(\Omega \right)\right\},\\ H_{0}^{1}\left(\Omega \right):=\left\{v:v\in H^{1}\;|\;v=0\;\mathrm{on}\;\partial \Omega \right\}. \end{array}$$

By multiplication of the first equation (1) with **v** ∈ *H*_0_^1^ and the second equation (2) with *q* ∈ *L* ^2^(*Ω*), subsequently integrating over the domain *Ω*, applying the Gauss theorem, and incorporating the boundary condition (3), we obtain the variational form.

Find **u** ∈ *H*_0_^1^(*Ω*) and *p* ∈ *L* ^2^(*Ω*) such that

(5) $$\begin{array}{c} a\left(u,v\right)-b\left(v,p\right)=F\left(v\right),\;\forall v\in H_{0}^{1}\left(\Omega \right),\\ b\left(u,q\right)=0,\;\forall q\in L^{2}\left(\Omega \right), \end{array}$$

where *a*(·, ·) and *b*(·, ·) are continuous bilinear forms defined as

(6) $$\begin{array}{c} a\left(u,v\right)=\int_{\Omega }\nabla u:\nabla v\;dx,\\ b\left(u,q\right)=\int_{\Omega }\left(\mathrm{div}v\right)q\;dx,\\ F\left(v\right)=\int_{\Omega }f\cdot v\;dx, \end{array}$$

where ∇**u** : ∇**v** represents a componentwise scalar product that is ∇*u*_*x*_ · ∇*v*_*x*_ + ∇*u*_*y*_ · ∇*v*_*y*_ and *a* : *H*_0_^1^(*Ω*) × *H*_0_^1^(*Ω*) → *ℛ* and *b* : *H*_0_^1^(*Ω*) × *L* ^2^(*Ω*) → *ℛ*. The well-posedness follows from the coercivity of *a*(·, ·) in the Lax-Milgram theorem [19, 20] and partly from the inf-sup condition [7, 8, 16, 21–23]. Below is a sketch of the analysis of the existence uniqueness and stability of the solution (**u**, *p*) ∈ **V** × *W* = *H*_0_^1^(*Ω*) × *L* ^2^(*Ω*) of mixed problem (5):

(i) the bilinear form *a*(·, ·) is bounded or continuous if
  (7) $$\begin{array}{c} \left|a\left(u,v\right)\right|\leq \alpha {\left\|u\right\|}_{V}{\left\|v\right\|}_{V},\;\forall u,v\in V,\;\;\alpha \in R; \end{array}$$
(ii) the bilinear form *a*(·, ·) is coercive on *V* : = *H*_0_^1^(*Ω*); that is, there exists a positive constant *α*_1_:
  (8) $$\begin{array}{c} a\left(v,v\right)\geq \alpha_{1}{\left\|v\right\|}_{V}^{2},\\ \forall v\in V=\text{ker}B=\left\{v\in V:b\left(v,q\right)=0\;\;\forall q\in W\right\}; \end{array}$$
(iii) the bilinear form *a*(·, ·) is symmetric and nonnegative if
  (9) $$\begin{array}{c} a\left(u,v\right)=a\left(v,u\right),\;\;a\left(v,v\right)\geq 0,\;\forall u,v\in V; \end{array}$$
(iv) the bilinear form *b*(·, ·) is bounded if
  (10) $$\begin{array}{c} \left|b\left(u,q\right)\right|\leq \alpha_{0}{\left\|u\right\|}_{V}{\left\|q\right\|}_{W},\;\forall u\in V,\;q\in W,\;\alpha_{0}\in R; \end{array}$$
(v) the bilinear form *b*(·, ·) satisfies the inf-sup condition; that is, there exists a constant *β*:
  (11) $$\begin{array}{c} \underset{0\neq q\in W}{\inf}\underset{\;\;0\neq v\in V}{\sup}\frac{\left|b\left(v,q\right)\right|}{{\left\|v\right\|}_{V}{\left\|q\right\|}_{W}}\geq \beta >0. \end{array}$$

For instance, in [22], it is shown that in our concrete case *b*(·, ·) fulfills the inf-sup condition; thus we can combine (i)–(v) to give the following theorem.

**Theorem 1 .**

The variational problem (5) is uniquely solvable provided properties (i)–(v) are all satisfied.

The proof relies on the closed range theorem and on the Lax-Milgram theorem. The details can be found in [22, 24].

### 2.1. The Mixed Finite Element Discretization

The mixed finite element discretization of the weak formulation of the Stokes equations produces a linear algebraic system of equations. The finite element method described here is based on [7, 16, 19, 21, 23, 25]. We will introduce the concept of mixed finite element methods. Details can be found in [7, 19–21, 25].

We assume that *Ω*⊆*ℛ* ^2^. We define the finite dimensional spaces. Let *W*_*h*_ and **V**_*h*_ be subspaces of *W* and **V**, respectively.

Now we can formulate a discrete version of problem (5).

Find a couple (**u**_*h*_, *p*_*h*_) ∈ **V**_*h*_ × *W*_*h*_ such that

(12) $$\begin{array}{c} a\left(u_{h},v_{h}\right)-b\left(v_{h},p_{h}\right)=F\left(v_{h}\right),\;\forall v_{h}\in V_{h},\\ b\left(u_{h},q_{h}\right)=0,\;\forall q_{h}\in W_{h}. \end{array}$$

The finite element discretization should satisfy the inf-sup condition. The following theorem shows that again the inf-sup condition is of major importance (for the proof we refer to [22]).

**Theorem 2 .**

Assume that *a* is *V*_*h*_-elliptic (with *h* independent ellipticity constant) and that there exists a constant *β* > 0 (independent of *h*) such that the discrete inf-sup condition

(13) $$\begin{array}{c} \underset{0\neq q_{h}\in W_{h}}{\inf}\underset{\;\;0\neq v_{h}\in V_{h}}{\sup}\frac{\left|b\left(v_{h},q_{h}\right)\right|}{{\left\|v_{h}\right\|}_{V_{h}}{\left\|q_{h}\right\|}_{W_{h}}}\geq \beta >0 \end{array}$$

holds. Then the associated (discretized, steady state) Stokes problem has a unique solution (**u**_*h*_, *p*_*h*_), and there exists a constant *β*_1_ such that

(14) u−uhV+p−phW ≤β1inf⁡v∈Vhu−uhV+inf⁡q∈Whp−phW.

If the basis of *W*_*h*_ is given by {*ψ*_1_,…, *ψ*_*m*_} and of *V*_*h*_ is given by {*φ*_1_,…, *φ*_*n*_}, then

(15) $$\begin{array}{c} u_{h}=\overset{n_{i}}{\underset{i=1}{\sum }}u_{i}\cdot \varphi_{i}+\overset{n_{i}+n_{\partial }}{\underset{i=n_{i}+1}{\sum }}u_{i}\cdot \varphi_{i},\\ p_{h}=\overset{m}{\underset{k=1}{\sum }}p_{k}\psi_{k}, \end{array}$$

where *n*_*i*_ is the number of inner nodes and *n*_∂_ is the number of boundary nodes so the coefficients **u**_*i*_ : *i* = *n*_*i*_ + 1,…, *n*_*i*_ + *n*_∂_ interpolate the boundary data and *n* = *n*_*i*_ + *n*_∂_. The mixed finite element entails partitioning of the solution domain *Ω* into quadrilaterals; in our case that is *Ω* = ∪_*i*_ *τ*_*i*_ we denote a set of rectangular (square) elements by *T*_*h*_ = {*τ*_1_, *τ*_2_, *τ*_3_,…} and on each element *τ*_*i*_ and we denote the space *P*_*k*_(*τ*_*i*_) of degree less than or equal to *k*. There are a variety of finite element pairs whose effectiveness is through stabilization [26]. In this work we are going to use Hoods-Taylor *Q*_2_-*Q*_1_ square finite elements which are known to be stable.

We specify

(16) $$\begin{array}{c} V_{h}:=\left\{u_{h}\in V{\left|u_{h}\right|}_{\tau_{i}}\in P_{2}\left(\tau_{i}\right),\forall \;\text{elements}\;\tau_{i}\right\},\\ W_{h}:=\left\{p_{h}\in W{\left|p_{h}\right|}_{\tau_{i}}\in P_{1}\left(\tau_{i}\right),\forall \;\text{elements}\;\tau_{i}\right\}. \end{array}$$

An element (**u**_*h*_, *p*_*h*_) ∈ *W*_*h*_ × *V*_*h*_ is uniquely determined by specifying *d* components of **u**_*h*_ on the nodes and on the midpoints of the edges of the elements and the values of *p*_*h*_ on the nodes of the elements. The mixed finite element method results in the coupled linear algebraic system which has to be solved by the appropriate solvers. The resulting system is

(17) $$\begin{array}{c} \left[\begin{array}{cc} A_{h} & B_{h}^{T}\\ B_{h} & O \end{array}\right]\left[\begin{array}{c} u_{h}\\ p_{h} \end{array}\right]=\left[\begin{array}{c} f_{h}\\ g_{h} \end{array}\right]; \end{array}$$

with *A*_*h*_ being a block Laplacian matrix and *B*_*h*_ being the divergence matrix whose entries are given by

(18) $$\begin{array}{c} A=\left[a_{ij}\right],\;a_{ij}=\int_{\Omega }{\left(\nabla \phi_{i}:\nabla \phi_{j}\right)}_{i,\;j=1,\;\ldots ,\;n},\\ B=\left[b_{ki}\right],\;b_{ki}=-\int_{\Omega }{\left(\psi_{k}\nabla \cdot \phi_{i}\right)}_{i=1,\;\ldots ,\;n;\;k=1,\;\ldots ,\;m}. \end{array}$$

The entries of the right hand side vector are

(19) $$\begin{array}{c} f=\left[f_{i}\right],\;f_{i}=\int_{\partial \Omega }f\cdot \phi_{i}-\overset{n+n_{\partial }}{\underset{i=n+1}{\sum }}u_{i}\int_{\Omega }\left(\nabla \phi_{i}:\nabla \phi_{j}\right),\\ g=\left[g_{k}\right],\;g_{k}=\overset{n+n_{\partial }}{\underset{i=n+1}{\sum }}u_{i}\int_{\Omega }\left(\psi_{k}\nabla \cdot \phi_{i}\right). \end{array}$$

The linear algebraic system can be represented as

(20) $$\begin{array}{c} Mx=b, \end{array}$$

where $ℳ:=\left[\begin{array}{cc} A_{h} & B_{h}^{T}\\ B_{h} & O \end{array}\right]$, $x:=\left[\begin{array}{c} u_{h}\\ p_{h} \end{array}\right]$, and $b:=\left[\begin{array}{c} f_{h}\\ g_{h} \end{array}\right]$.

The solution vectors (**u**_*h*_, *p*_*h*_) from (15) are the mixed finite element solution. The system (17)–(19) is called the discrete Stokes problem.

The discretization and assembling of matrices are done by the MATLAB implementation of the mixed finite element method [8]. *A*_*h*_ is stiffness matrix resulting from the discretization of the Laplacian. The resultant coefficient matrix is large, sparse, indefinite and the system must be solved iteratively, in this case by multigrid solvers. The multigrid solver is a well known fast solver for the elliptic partial differential equations [2, 5].

## 3. Multigrid Method

The main focus of this section is the construction of the multigrid solver to find the approximate solution of (20) at the finest mesh/discretization. Let (**V**_*l*_ × *W*_*l*_) be a sequence of subspaces of the finite dimensional subspaces *W*_*h*_ and *V*_*h*_ defined on sequence of grids *l* ∈ {0,1, 2,3,…, *l*_max⁡_} with mesh sizes *h*_0_, *h*_1_, *h*_2_,…, *h*_*l*_ with *h*_*l*+1_ : = (1/2)*h*_*l*_. We define a hierarchy/family of nested finite element subspaces for the velocity and pressure:

(21) Vl×Wl⊂Vl+1×Wl+1⊂Vh×Wh⊂V×W=H01Ω×L2Ω,

where (*V*_*l*+1_ × *W*_*l*+1_) subspace which corresponds to *Ω*_*l*+1_ is the refinement of *Ω*_*l*_ with subspace (*V*_*l*_ × *W*_*l*_) such that *Ω*_*l*_ ⊂ *Ω*_*l*+1_ ⊂ *Ω*. At the discrete level with the defined discrete spaces and bases, the linear algebraic system is defined by

(22) $$\begin{array}{c} M_{l}x_{l}=b_{l}, \end{array}$$

where ${ℳ}_{l}:=\left[\begin{array}{cc} A_{l} & B_{l}^{T}\\ B_{l} & O \end{array}\right]$, *x*_*l*_ : = [**u**_*l*_ *p*_*l*_], and $b_{l}:=\left[\begin{array}{c} f_{l}\\ g_{l} \end{array}\right]$.

The main goal is to find the pair *x*_*l*_ = (**u**_*l*_, *p*_*l*_) of the discrete velocity and the discrete pressure variables at the finest level *l*.

Now we introduce the multigrid iteration for solving the discretized equation (22) on grid *l*. We define the multigrid algorithm at level *l* as MGM_*l*_(**u**_*l*_^new^, *p*_*l*_^new^, **u**_*l*_^old^, *p*_*l*_^old^, **f**_*l*_, *g*_*l*_, *m*_1_, *m*_2_), where

- (**u**_*l*_^new^, *p*_*l*_^new^) is the output of velocity and pressure after one step of the multigrid algorithm at level *l*;
- **u**_*l*_^old^ is the input velocity at level *l*;
- *p*_*l*_^old^ is the input pressure at level *l*;
- *R*_**u**,*l*,*l*−1_ and *R*_*p*,*l*,*l*−1_ are restriction operators for velocity and pressure, respectively, from level *l* to level *l* − 1;
- *P*_**u**,*l*−1,*l*_ and *P*_*p*,*l*−1,*l*_ are prolongation operators for velocity and pressure, respectively, from level *l* − 1 to level *l*.

**Algorithm 3 (multigrid algorithm).**

(23) $$\begin{array}{c} {\text{MGM}}_{l}\left(u_{l}^{\text{new}},p_{l}^{\text{new}},u_{l}^{\text{old}},p_{l}^{\text{old}},f_{l},g_{l},m_{1},m_{2}\right) \end{array}$$

if *l* = 0 (coarsest grid)

(24) AlBlTBlOulnewplnew =flgl =MGMlulnew,plnew,ulold,plold,fl,gl,m1,m2

else *l* > 0 define MGM_*l*_(**u**_*l*_^new^, *p*_*l*_^new^, **u**_*l*_^old^, *p*_*l*_^old^, *g*_*l*_, **f**_*l*_, *m*_1_, *m*_2_)

 (1)  Pre-Smoothing: Smoothing operator *𝒮* starting with, (**u**_*l*_^old^, *p*_*l*_^old^) with *m*_1_ smoothing steps, producing $\left({\tilde{u}}_{l},{\tilde{p}}_{l}\right)$,
  (25) $$\begin{array}{c} \left[\begin{array}{c} {\tilde{u}}_{l}\\ {\tilde{p}}_{l} \end{array}\right]=\left[\begin{array}{c} u^{\text{old}}\\ p^{\text{old}} \end{array}\right]-S^{-1}\left(\left[\begin{array}{cc} A_{l} & B_{l}^{T}\\ B_{l} & O \end{array}\right]\left[\begin{array}{c} u_{l}^{\text{old}}\\ p_{l}^{\text{old}} \end{array}\right]-\left[\begin{array}{c} f_{l}\\ g_{l} \end{array}\right]\right) \end{array}$$
  (a)  defect/residual
    (26) $$\begin{array}{c} r_{l}=f_{l}-\left(A_{l}{\tilde{u}}_{l}+B_{l}^{T}{\tilde{p}}_{l}\right),\\ d_{l}=g_{l}-B_{l}{\tilde{u}}_{l} \end{array}$$
(2)  restrict the defect
  (27) $$\begin{array}{c} r_{l-1}=R_{u,l,l-1}r_{l},\\ d_{l-1}=R_{p,l,l-1}d_{l}, \end{array}$$
(3)  approximate solution
  (28) $$\begin{array}{c} \left[\begin{array}{cc} A_{l-1} & B_{l-1}^{T}\\ B_{l-1} & O \end{array}\right]\left[\begin{array}{c} {\tilde{v}}_{l-1}\\ {\tilde{q}}_{l-1} \end{array}\right]=\left[\begin{array}{c} r_{l-1}\\ d_{l-1} \end{array}\right] \end{array}$$
(4)  applying one/two iterations of MGM_*l*−1_ at the recursive call:
  (a)  apply *μ* steps of MGM_*l*−1_
  (b)  Set **v**_*l*−1_^0^ = 0
  (c)  Set *q*_*l*−1_^0^ = 0
  (d)  compute
   for *μ* = 1 : 2
    (29) v~l−1,q~l−1 =MGMl−1v~l−1,q~l−1,vl−10,ql−10,rl−1,dl−1,m1,m2;
   end
(5)  *Correction Step* define the new iterate:
  (30) $$\begin{array}{c} u_{l}^{*}:={\tilde{u}}_{l}-P_{u,l-1,l}{\tilde{v}}_{l-1},\\ p_{l}^{*}:={\tilde{p}}_{l}-P_{p,l-1,l}{\tilde{q}}_{l-1}. \end{array}$$

*Postsmoothing*. Starting with (**u**_*l*_^∗^, *p*_*l*_^∗^) perform *m*_2_ smoothing steps using smoothing operator *𝒮* to produce (**u**_*l*_^new^, *p*_*l*_^new^):

(31) $$\begin{array}{c} \left[\begin{array}{c} u^{\text{new}}\\ p^{\text{new}} \end{array}\right]=\left[\begin{array}{c} {\tilde{u}}_{l}^{*}\\ {\tilde{p}}_{l}^{*} \end{array}\right]-S^{-1}\left(\left[\begin{array}{cc} A_{l} & B_{l}^{T}\\ B_{l} & O \end{array}\right]\left[\begin{array}{c} {\tilde{u}}_{l}^{*}\\ {\tilde{p}}_{l}^{*} \end{array}\right]-\left[\begin{array}{c} f_{l}\\ g_{l} \end{array}\right]\right). \end{array}$$

The multigrid method described above belongs to a class of optimal order methods for solving linear systems emanating from the discretization techniques like the finite element method. Its known convergence speed does not deteriorate when the discretization is refined whereas classical iterative solvers slow down for the decreasing mesh size [1, 2, 5, 6]. The starting point of the multigrid concept is the observation that classical iteration methods have some smoothing properties. The operator *𝒮* represents such methods; in this study it represents Braess-Sarazin, inexact Uzawa, distributed Gauss Seidel, and the preconditioned minimum residual method. These methods are characterized by poor/slow global convergence rates and for errors whose length scales are comparable to mesh sizes, they provide rapid damping leaving behind smooth, longer wave length errors. These smooth parts of the error are responsible for the poor convergence. A geometric multigrid method involves a hierarchy of meshes and related discretization. A quantity that is smooth on a certain grid can also be approximated on a coarser grid. Low frequency error components can be effectively reduced by a coarse grid correction procedure. Since the action of a smoothing iteration leaves only smooth error components, it is possible to represent them as the solution of an appropriate coarser system. Once this coarser problem is solved, the solution is interpolated back to the fine grid to correct the fine approximation for its low frequency errors. The most essential ingredients of the multigrid method are the smoothing operator, for which using a wrong smoother will destroy the efficiency of the entire multigrid method, and the coarse grid correction which involves the prolongation and the restriction operators. In multigrid methods we have to transform information from one grid to another and for that purpose we use prolongations and restrictions operators. Restriction transfers values from fine grid to the next coarse grid. Prolongation transfers values from the coarse grid to the next fine grid.

Next we discuss the key components of the multigrid method.

(a) Intergrid transfer operators: the intergrid transfer operators are the restriction and prolongation between different grid levels. The restriction operator maps the residual from the finer grid to a coarser grid while the prolongation operator transfers vectors from coarse grid to fine grid. The restriction between levels *l* and *l* − 1 is defined by
  (32) $$\begin{array}{c} R_{\left(l,l-1\right)}:=\left(\begin{array}{cc} R_{\left(u,l,l-1\right)} & 0\\ 0 & R_{\left(p,l,l-1\right)} \end{array}\right), \end{array}$$
  where the restriction operators *R*_(**u**,*l*,*l*−1)_ : *ℛ* ^*n*_*l*_^ → *ℛ* ^*n*_*l*_−1^ and *R*_(*p*,*l*,*l*−1)_ : *ℛ* ^*m*_*l*_^ → *ℛ* ^*m*_*l*−1_^ for velocity and pressure, respectively. The prolongation between levels *l* − 1 and *l* is defined again as
  (33) $$\begin{array}{c} P_{\left(l-1,l\right)}:=\left(\begin{array}{cc} P_{\left(u,l-1,l\right)} & 0\\ 0 & P_{\left(p,l-1,1\right)} \end{array}\right), \end{array}$$
  where the prolongation operators *P*_(**u**,*l*−1,*l*)_ : *ℛ* ^*n*_*l*−1_^ → *ℛ* ^*n*_*l*_^ and *P*_(*p*,*l*−1,*l*)_ : *ℛ* ^*m*_*l*−1_^ → *ℛ* ^*m*_*l*_^ are representations of the following relations **V**_*l*−1_ ⊂ **V**_*l*_ for the quadratic interpolation of the velocity (*Q*_2_) and *W*_*l*−1_ ⊂ *W*_*l*_ for the linear interpolation of the pressure (*Q*_1_).
(b) Coarse grid correction: the other key ingredient of the multigrid method is the coarse grid correction. In the multigrid solution process we need to solve the problem at the finest define level *l* = *l*_max⁡_. The problem is defined on the coarser grid levels and on the coarsest grid level the problem is solved exactly. There are very few situations in which a grid can be coarsened to the extent that it is not practical to solve the problem using a direct method but iteratively. In this work the iterative solver used as a smoother is applied to solve the problem at the coarsest level.

### 3.1. The Smoothers

The most crucial part is the proper choice of a smoothing technique. Usually, the well-known smoothing iterations for the scalar problems (damped Jacobi or Gauss-Seidel relaxation) are not appropriate for saddle point problems or are even not defined, for example, in saddle point systems like (22). There are natural ways to generalize scalar smoothing schemes to systems of PDEs. The smoothing process is the main ingredient of the multigrid method. The convergence of the multigrid method is influenced by the smoothing process [11, 14, 18]. We perform a number of iterations of an iterative solver to smooth the residual. The main goal is to compare the effectiveness of different iterative schemes as smoothers of the multigrid methods. On each level of a multigrid method, a system involving operator *𝒮* has to be solved approximately. The smoother dumps out highly oscillating error modes of the systems. In this paper we consider the following smoothing process:

(34) $$\begin{array}{c} \left(\begin{array}{c} u_{l}^{i+1}\\ p_{l}^{i+1} \end{array}\right)=\left(\begin{array}{c} u_{l}^{i}\\ p_{l}^{i} \end{array}\right)-S_{l}^{-1}\left[\left(\begin{array}{cc} A_{l} & B_{l}^{T}\\ B_{l} & O \end{array}\right)\left(\begin{array}{c} u_{l}^{i}\\ p_{l}^{i} \end{array}\right)-\left(\begin{array}{c} f_{l}\\ 0 \end{array}\right)\right]. \end{array}$$

Several smoothers have been proposed and applied in literature. Brandt [4] advocates for the use of the distributed Gauss Seidel smoothing. The Vanka-type smoother is widely used with a coupled Gauss Seidel scheme [13, 14] that introduces the idea of transforming smoothers and combines with incomplete factorization to develop an efficient smoothing. John and Tobska [14] and Pernice [15] used the Braess-Sarazin-type smoother with the Schur complement schemes as smoothers which exhibit wonderful smoothing properties. The following algorithms describe the iterative schemes that are used as smoothers in this study.

#### 3.1.1. Braess-Sarazin-Type Smoother

The Braess-Sarazin smoothers proposed in [17] and used in [13, 18] solve a large saddle point problem in each smoothing step. This Braess-Sarazin or SIMPLE-type iteration uses $\left(\begin{array}{cc} \hat{A} & B^{T}\\ B & O \end{array}\right)$ as a smoother for the saddle point problem (22). The smoother as presented in [17] and generalised in [18] consisted of constant application of the smoothing iteration:

(35) $$\begin{array}{c} \left(\begin{array}{c} u_{l}^{i+1}\\ p_{l}^{i+1} \end{array}\right)=\left(\begin{array}{c} u_{l}^{i}\\ p_{l}^{i} \end{array}\right)-{\left(\begin{array}{cc} {\hat{A}}_{l} & B_{l}^{T}\\ B_{l} & O \end{array}\right)}^{-1}\left[\left(\begin{array}{cc} A_{l} & B_{l}^{T}\\ B_{l} & O \end{array}\right)\left(\begin{array}{c} u_{l}^{i}\\ p_{l}^{i} \end{array}\right)-\left(\begin{array}{c} f_{l}\\ g_{l} \end{array}\right)\right] \end{array}$$

with ${\hat{A}}_{l}=\alpha \mathrm{diag}(A_{l})$ and *α* = 2 given. The smoothing Braess-Sarazin iteration (35) solves the auxiliary problem

(36) $$\begin{array}{c} \left(\begin{array}{cc} \alpha {\hat{A}}_{l} & B_{l}^{T}\\ B_{l} & O \end{array}\right)\left(\begin{array}{c} {\hat{u}}_{l}^{i}\\ {\hat{p}}_{l}^{i} \end{array}\right)=\left(\begin{array}{c} r_{l}^{i}\\ s_{l}^{i} \end{array}\right) \end{array}$$

with **r**_*l*_^*i*^ = *A*_*l*_ **u**_*l*_^*i*^ + *B*_*l*_^*T*^ *p*_*l*_^*i*^ − **f**_*l*_ and *s*_*l*_^*i*^ = *B*_*l*_ **u**_*l*_^*i*^ − *g*_*l*_. Inherent in the system system (36) is the problem of the auxiliary pressure variable ${\hat{p}}_{l}$

(37) $$\begin{array}{c} {\hat{S}}_{l}{\hat{p}}_{l}=B_{l}{\hat{A}}_{l}^{-1}r_{l}^{i}-\alpha B_{l}u_{l}^{i}. \end{array}$$

This system is solved approximatively by an iterative solver. From the system we get ${\hat{p}}_{l}$ approximately which can be used to approximately determine ${\hat{u}}_{l}$ from

(38) $$\begin{array}{c} \alpha {\hat{A}}_{l}{\hat{u}}_{l}=r_{l}^{i}-B_{l}^{T}p_{l}^{i}. \end{array}$$

#### 3.1.2. Inexact Uzawa Type Smoothers

The variant of the inexact Uzawa iteration used as a smoother is outlined.

**Algorithm 4 .**

(1) For *i* = 1: smoothing steps.

(2) Compute the residual **r**_*i*_ = **f** − *A* **u**_*i*_ − *B* ^*T*^ *p*_*i*_.

(3) Compute the residual *s*_*i*_ = *g* − *B* **u**_*i*_.

(4) Solve $\tilde{A}w_{i}=r_{i}$.

(5) Solve $\tilde{S}d_{i}=B^{T}w_{i}-s_{i}$.

(6) Solve $\tilde{A}w_{i}=r_{i}-Bd_{i}$.

(7) Update the velocity and pressure

(39) $$\begin{array}{c} \left(\begin{array}{c} u_{i+1}\\ p_{i+1} \end{array}\right)=\left(\begin{array}{c} u_{i}\\ p_{i} \end{array}\right)+\left(\begin{array}{c} w_{i}\\ d_{i} \end{array}\right); \end{array}$$

End.

Step (6) in the outline may be rearranged as $w_{i}:=w_{i}-{\tilde{A}}^{-1}(Bd_{i})$ with ${\tilde{A}}^{-1}(Bd_{i})$ obtained as a by-product of step (5). This saves the application of ${\tilde{A}}^{-1}$ at the end of every outer iteration and hence improves the efficiency of the algorithm. The other variants of the inexact Uzawa method are analysed in [26–28].

#### 3.1.3. The Distributed Gauss Seidel Type Smoothers (DGS)

The standard smoothing iteration schemes like Jacobi and Gauss Seidel smoothers are not applicable to the system (22) because of the nature of the coefficient matrix; particularly the zero block in the diagonal hampers the smoothing process. The distributive smoother transforms the vital operators to the main diagonal and applied as a decoupled smoother. The DGS was introduced in [4] is related to a successive application of standard Gauss Seidel applied to the matrix operator *ℳ* (22) and $𝒢=\left(\begin{array}{cc} I_{l} & B_{l}^{T}\\ O & -B_{l}B_{l}^{T} \end{array}\right)$ with $ℳ𝒢=\left(\begin{array}{cc} A_{l} & B_{l}^{T}\\ B_{l} & B_{l}B_{l}^{T} \end{array}\right)$. We solve the transformed residual equation

(40) $$\begin{array}{c} \left(\begin{array}{cc} {\hat{A}}_{l} & B_{l}^{T}\\ B_{l} & {\hat{A}}_{p} \end{array}\right)\left(\begin{array}{c} w_{i}\\ q_{i} \end{array}\right)=\left(\begin{array}{c} r_{u}\\ r_{p} \end{array}\right) \end{array}$$

with $\hat{A}$ and ${\hat{A}}_{p}$ being invertible approximations of *A* and *A*_*p*_ : = *BB* ^*T*^, respectively. A single iteration with the update through a distributive matrix *𝒢* is performed by the following algorithm.

**Algorithm 5 ([u ^*i*+1^, *p* ^*i*+1^] ← DGS(u ^*i*^, *p* ^*i*^)).**

(1)   Smooth momentum equations

(41) $$\begin{array}{c} w=u^{i}+{\hat{A}}^{-1}\left(f-Au^{i}-B^{T}p^{i}\right). \end{array}$$

(2) Smooth the transformed continuity equation

(42) $$\begin{array}{c} q={\hat{A}}_{p}^{-1}\left(g-Bw\right). \end{array}$$

(3) Transform the correction back to the original variables

(43) $$\begin{array}{c} u^{i+1}=w+B^{T}q,\\ p^{i+1}=p^{i}-BB^{T}q. \end{array}$$

The DGS has been widely used as a smoother for the finite difference discretization. In this paper the DGS type smoothers are used for finite element discretization of the Stokes problem.

#### 3.1.4. The Preconditioned Minimum Residual Smoother

The preconditioned minimum residual method is a Krylov subspace method for solving symmetric indefinite systems and uses popular block preconditioners. This method is used as a smoother for the multigrid method of the Stokes problem in this paper. For the Stokes equations, the classical block-diagonal preconditioner for MINRES method [8] is

(44) $$\begin{array}{c} P=\left(\begin{array}{cc} \hat{A} & 0\\ 0 & \hat{S} \end{array}\right) \end{array}$$

with $\hat{S}=B{\hat{A}}^{-1}B^{T}$. The block preconditioning requires the solution of two systems of equations with matrices $\hat{A}$ and $\hat{S}$ at each MINRES iteration. If *P* ^−1^ is computed exactly, the preconditioned Krylov methods converge in two or three steps [10]. For practical implementations, the Schur complement $\hat{S}$ is replaced by the mass matrix *M*_*p*_ of the pressure space. For discontinuous pressure space, *M*_*p*_ is block diagonal and easy to invert. For continuous pressure space, say *Q*_1_, the mass matrix *M*_*p*_ can be further replaced by its diagonal matrix [8].

### 3.2. Multigrid Convergence

The convergence analysis of the multigrid method relies on the two properties, namely, the approximation and the smoothing. The general convergence rates are independent of *h* (the mesh size), *l* is the level of discretization, and *m*_1_ and *m*_2_ are the number of pre- and postsmoothing iterations [1, 12, 18]. The results for the convergence of the multigrid method for the scalar elliptic problems cannot apply to the Stokes equations. We provide a snapshot of the available convergence results of the multigrid method for Stokes equations. The ideas presented in this paper are based on the work in [12, 16, 18]. An iteration of single multigrid step consists of a combination of smoothing step and a coarse grid correction step. We will consider the multigrid convergence with the Braess-Sarazin smoother with *𝒮*_*l*_ being the iteration matrix of the smoother (34) and the *ℳ*_*l*_ being the Stokes stiffness matrix in (22). The operator *P*_*l*_ and its adjoint *R*_*l*_ are intergrid transfer operators, prolongation, and the restriction, respectively. The convergence analysis of the multigrid method begins with the analysis of the two-grid method, with *m*_1_ and *m*_2_ the pre- and post-smoothing steps, respectively, applied to (22) results in the iteration matrix

(45) $$\begin{array}{c} L_{l}=S_{l}^{m_{1}}\left(I_{l}-P_{l}M_{l-1}^{-1}R_{l}M_{l}\right)S_{l}^{m_{2}}. \end{array}$$

The key point on the analysis of the multigrid method is that the error can be split into two components. That is the one produced by the smoothing process and the one produced by the coarse grid correction. The coarse grid error consists of the low frequency components and the smoothing consists of the high frequency components of error. The ability to cope with the low frequency components is called the approximation property and with the second is called the smoothing property. For the analysis of the multigrid convergence [2, 29] used the framework based on the smoothing and approximation property. For analysis we define the following norms, Euclidean norm by ‖·‖, and on *ℛ* ^*n*_*l*_+*m*_*l*_^ the following norm is applied:

(46) ulplh2:=ul2+hl2pl2=Θlulplh2hhwith  Θl:=InlOOIml.

Furthermore we introduce

(47) $$\begin{array}{c} {\hat{M}}_{l}:=\Theta_{l}^{-1}M_{l}\Theta_{l}^{-1},\;\;{\hat{S}}_{l}:=\Theta_{l}^{-1}S_{l}\Theta_{l}^{-1}. \end{array}$$

Using the norms defined above and taking *m*_1_ = *m* and *m*_2_ = 0 above we obtain

(48) Llh=ΘlMl−1−PlMl−1−1RlΘlΘl−1LlSlmΘl−1≤ΘlMl−1−PlMl−1−1RlΘlM^lS^l.

The theorems below state the two properties and the multigrid convergence. For detailed proof we refer to [12, 18].

**Theorem 6 (approximation property).**

Assume that *Ω* is such that the problem (5) is *H* ^2^-regular. Let *ℳ*_*l*_ be the coefficient stiffness matrix and *R*_*l*_ and *P*_*l*_ the prolongation and the restriction operators. Then there exists a constant *C*_*ℳ*_ independent of *l* and using *h*-scaling induced by *ℳ*_*l*_ then

(49) $$\begin{array}{c} {\left\|\Theta_{l}\left(M_{l}^{-1}-P_{l}M_{l-1}^{-1}R_{l}\right)\Theta_{l}\right\|}_{h}\leq C_{M}{\left\|M_{l}^{-1}\right\|}_{2}, \end{array}$$

where *C*_*ℳ*_ = *Ch* ^2^.

The smoothing property is dependent on the smoother used. It varies from one smoother to another. In this work we used the Braess-Sarazin in which we solve the system (37) exactly and sufficiently accurate inexact inner solver.

**Theorem 7 (smoothing property).**

Let *ℳ*_*l*_ be the coefficient stiffness matrix and the smoothing operator *𝒮*_*l*_. Then

(50) $$\begin{array}{c} \left\|{\hat{M}}_{l}{\hat{S}}_{l}^{m}\right\|\leq g\left(m\right)\left\|M_{l}\right\|, \end{array}$$

where *g*(*m*) = *ch*_*l*_^2^/(*m* − 1) for *m* ≥ 2 and *g*(*m*) is a decreasing function with lim_*m*→*∞*_⁡*g*(*m*) = 0.

Combining the approximation property Theorem 6 with the smoothing property Theorem 7 produces a two-grid convergence result.

**Theorem 8 .**

Assume that *m*_2_ = 0 and that *Ω* is such that the problem (5) is *H* ^2^-regular. Then for the two-grid method the following holds:

(51) $$\begin{array}{c} {\left\|M_{l}\right\|}_{h}\leq \frac{C_{M}}{m-1},\;m\geq 2 \end{array}$$

with a constant *C*_*ℳ*_ independent of l and m.

Using this two-grid contraction number bound the multigrid *W*-cycle method convergence results can be derived using ideas in [1, 2].

## 4. Numerical Results

In this section we present the numerical solution of classical Stokes problem (1)–(3) using the solver presented above. The solver is denoted by MGM (Algorithm 3). We present the results of this method as outlined above to run the traditional test problem, the driven cavity flow problem [11, 12, 27, 28]. It is a model of the flow in a square cavity (the domain is *Ω*_□_) with the top lid moving from left to right in our case the regularized cavity model {*y* = 1 : −1 ≤ *x* ≤ 1∣*u*_*x*_ = 1 − *x* ^4^} [11]. The Dirichlet no-slip boundary condition is applied on the side and bottom boundaries. The mixed finite element method was used to discretize the cavity domain *Ω* = (−1,1)^2^.

We pay particular attention to the computational performance of the multigrid method on the system (22) at different grid levels. We compare the effectiveness of different smoothing/relaxation methods in the performance of the multigrid method and different approximations for the preconditioners $\hat{A}$ and $\hat{S}$ of the smoothers. The following setup of the smoothers listed is considered.

- Distributed Gauss Seidel (DGS) smoother: we use one Gauss Seidel iteration for the evaluations of $\hat{A}$ and one Gauss Seidel iteration for the computation of ${\tilde{A}}_{p}$. The method becomes DGSMG.
- Inexact Uzawa smoother (IUzawa): the two cases are considered for the evaluation of the preconditioners. Firstly, the approximation $\hat{A}=\mathrm{diag}(A)$ and one *v*(1,1)-cycle is used to approximate Schur compliment matrix $B\hat{A}B^{T}$. The second case is to use one *v*(1,1)-cycle for both evaluations of $\hat{A}$ and $B\hat{A}B^{T}$. The method becomes IUZAWAMG.
- Braess-Sarazin smoother (B-S): the two cases are conspired for the evaluation of the preconditioners. Firstly, the approximation $\hat{A}=\mathrm{diag}(A)$ and one *v*(1,1)-cycle is used to solve the approximate Schur compliment matrix $B\hat{A}B^{T}$. The second case is to use one *v*(1,1)-cycle for both evaluations of $\hat{A}$ and $B\hat{A}B^{T}$. The method becomes B-SMG.
- PMINRES smoother: the first case is to use diagonal preconditioner for $\hat{A}$ and $\hat{S}$ and the second case is one *v*(1,1)-cycle for committing the inversion of the Laplacian operator for velocity as one *v*(1,1) cycle is used to approximate the Schur compliment using the pressure mass matrix to accelerate the MINRES. The method becomes PMINRESMG.

The comparison is made on the performance of the multigrid schemes with different smoothers (i)–(iv) and cases involving different approximations of preconditioners in terms of iterative counts and CPU time. The numerical treatment is given to the discrete Stokes problem which resulted from the mixed finite Hood-Taylor stable elements consisting of biquadratic elements for the velocities and bilinear elements for the pressure, on a uniform grid. Implementation of our algorithms was performed on a Windows 7 platform with 2.13 GHz speed intel dual core processor by using MATLAB 7.14 programming language and the MATLAB built-in Minres functions are used for the smoother. For the discretization we start with a uniform square grid with *h*_0_ = 1/2 and we apply regular refinements to this starting discretization to obtain the finest grid level. The discretized equations are solved using the multigrid iteration with the *W*-cycle and *V*-cycle and *m*_1_ and *m*_2_ being presmoothing and postsmoothing steps, respectively. The smoothers are determined by specifying approximations $\hat{A}$ and $\hat{S}$ as highlighted in (i)–(vi) and in all cases where one *v*-cycle inner multigrid iteration is used with *n*_1_ and *n*_2_ being Gauss Seidel iteration steps of the presmoothing and the postsmoothing, respectively.

In this work we use the structured mesh and regular refinements. The finite element matrices on the rectangular grids are assembled and the meshes are generated by the MATLAB IFISS toolbox [3] in a hierarchy of grids which are produced by successive regular refinements. We need to choose the coarse mesh (the starting mesh), the finest mesh which corresponds to the maximum level of refinement on which the final approximate solution is considered. The assembled matrices are stored for each refinement level for the system (22).  Table 1 shows an example of the refinement levels, we use the coarsest (starting) level to have 9 nodes for velocity and 4 nodes for pressure variables (level 1) but we start the computation at level 2.

Table 1 shows the refinement levels and the number of grid points (nodes) for each level.

The zero initial guess is chosen for all the tests. In all the tests the iterations are repeated until the tolerance ‖*R*_*i*_‖/‖*R*_0_‖ < 10^−6^, where $R_{i}=\left(\begin{array}{c} f_{i}\\ g_{i} \end{array}\right)-\left(\begin{array}{cc} A_{l} & B_{l}^{T}\\ B_{l} & O \end{array}\right)\left(\begin{array}{c} u_{i}\\ p_{i} \end{array}\right)$ is satisfied. The schemes converge if the stopping criteria are satisfied. The results show the first case of the evaluation, the preconditioners of the smoothers $\hat{A}$ and $\hat{S}$ with $\hat{A}=2\mathrm{diag}(A)$, and for all cases evaluation of $\hat{S}$ by one *v*-cycle inner multigrid iteration with *n*_1_ and *n*_2_ being Gauss Seidel iteration of the presmoothing and postsmoothing steps, respectively.

Tables 2 and 3 show the number of iterations and computing time to demonstrate the effects of different *V*-cycle (1, 2, 3, and 4) and *W*-cycle (1, 2, 3, and 4) being pre- and postsmoothing steps with Braess-Sarazin (B-S) smoother (diag(*A*), *v*(1,1)). We compare the performance of the *V*-cycle and *W*-cycle multigrid iterations with various smoothing steps at different grid levels using one of the smoothers, Braess-Sarazin.

From Tables 2 and 3 we observe that the number of iterations decreases when the smoothing steps decrease and the CPU time increases as expected with the increase in smoothing steps.

Tables 4 and 5 show the numerical results obtained of the multigrid solver at different grid levels. The number of *V*-cycle and *W*-cycle multigrid iterations and CPU time are shown, respectively. All the results presented underline the efficiency of the multigrid solver to indefinite systems of equations. In both tables we present results of the four studied smoothers of the multigrid solver. In Tables 4 and 5 we choose the approximation of the smoother preconditioners as $\hat{A}=2\mathrm{diag}(A)$ and $\hat{S}=v\text{-cycle}(1,1)$ for Braess-Sarazin, IUzawa, and PMINRES. For the DGS we use the one Gauss Seidel for both *A* and *A*_*p*_. We fix the number of smoothing steps to (3,3) for all the results in the tables.

Comparing the performance of the *V*-cycle and *W*-cycle multigrid solver, we observe that the smoothers have different effects on the performance of the multigrid solver. The multigrid solver is optimal and the iterations are bound for all the grid levels. In Tables 4 and 5 we compare all smoothers and we observe that the Braess-Sarazin smoother leads to faster convergence of the multigrid with fewer iterations and less CPU ahead of other smoothers. The inexact Uzawa did not disappoint in relaxing the error but the DGS and the PMINRES lead to more iterations and computing times. The other observation in Tables 4 and 5 is that the *W*-cycle converges in fewer iterations than the *V*-cycle though it has more computing times for all smoothers.

In Tables 6 and 7 we use different approximations for the preconditioner of the smoothers of the multigrid *V*-cycle and *W*-cycle, respectively. In applying the preconditioners, we approximate the preconditioner $\hat{A}$ of the Laplacian stiffness and sparse matrix *A* and $\hat{S}$ by a geometric multigrid *v*(1,1)-cycle method (*A*_*mg*_). The multigrid is a well-known fast solver for such systems. The multigrid solver is an inner iteration of the smoothers. The results in Tables 6 and 7 also show that the one iteration of the multigrid *v*-cycle is a suitable approximation of the smoothers since the multigrid solver has improved iterations from the ones in Tables 4 and 5. In both tables the multigrid method is optimal in solving the indefinite systems and the number of iterations is bounded for all smoothers independent of the mesh size or grid level.

Table 8 shows the changes in the estimated a posteriori errors for regularized driven cavity flow using *Q*_2_-*Q*_1_ approximation for the flow: using the strategy built in IFISS [3, 8] that, for every element error, the local error estimation is given by the combination of the energy norm of the velocity error and the *L*_2_ norm of the divergence error; that is,

(52) $$\begin{array}{c} \eta_{T}^{2}:={\left\|\nabla e_{T}\right\|}_{T}^{2}+{\left\|R_{T}\right\|}_{T}^{2}, \end{array}$$

where **e**_*T*_ is the velocity error estimate and *R*_*T*_ = ‖∇·**u**‖_*T*_ and *η* : = (∑_*T*∈*T*_*h*__ *η*_*T*_^2^)^1/2^ are the global error estimator, using different smoothers from one level to the other.

From Table 8 we note that the velocity divergence is clearly converging at a faster rate to *O*(*h* ^3^), which means that the estimated global error *η* is increasingly dominated by the velocity error component as *h* → 0.

**Figure 1:**
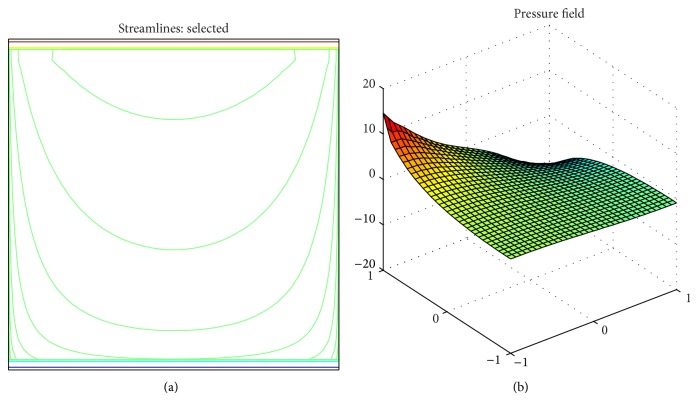
Velocity streamlines (a) and pressure plot (b) of the Stokes equation at level 5.

Figure 1 shows the sample grid output at the levels 1/64 and 1/128, the sample velocity solution (exponential streamlines), and the pressure plot at the same level with the same smoother.

## 5. Conclusion

The purpose of this study was to explore the multigrid solver for the Stokes equations. We have introduced four smoother iterative methods for both multigrids *V*-cycle and *W*-cycle to solve the indefinite systems emanating for the mixed finite element discretization of the Stokes problem. We analyse the construction of the multigrid solver, construction of the smoothers, computation costs, and CPU time as an indicator of the performance of each smoother at all grid levels. Numerical experimental results are given for both *V*-cycle and *W*-cycle for the smoothers at different grid levels. We have found out that for both cases and for all smoothers used in this study the multigrid solver is optimal and the number of iterations is bounded for all the grid levels. For the steady Stokes equations and the choices of the smoothers used the Braess-Sarazin like smoother became the best iteration to relax the error of the multigrid solver. All the numerical results show that the one *v*-cycle multigrid iteration is also a suitable preconditioner for the smoothers used.

## Figures and Tables

**Table 1:** Refinement levels and number of nodes (*n*_*l*_: number of velocity unknowns (×2) and *m*_*l*_: number of pressure unknowns).

Refinement level (*l*)	1	2	3	4	5	6	7	8

Mesh size (*h*_*l*_)	$\frac{1}{2}$	$\frac{1}{4}$	$\frac{1}{8}$	$\frac{1}{16}$	$\frac{1}{32}$	$\frac{1}{64}$	$\frac{1}{128}$	$\frac{1}{256}$

Velocity nodes (*n*_*l*_)	9	25	81	289	1089	4425	16641	66049
Pressure nodes (*m*_*l*_)	4	9	25	81	289	1089	4425	16641

**Table 2:** Number of iterations and CPU time for Braess-Sarazin (2diag(*A*), *v*-cycle(1,1)) multigrid *V*-cycle at different levels of refinement, tolerance = 10^−6^.

Levels	MG-*V*-cycle			
	*v*(1,1)	*v*(2,2)	*v*(3,3)	*v*(4,4)
	Iter(cpu time (sec))	Iter(cpu time (sec))	Iter(cpu time (sec))	Iter(cpu time (sec))
$\frac{1}{16}$	21 (7.1*e* − 02)	15 (7.3*e* − 02)	9 (2.1*e* − 02)	6 (8.6*e* − 02)
$\frac{1}{32}$	22 (5.4*e* − 01)	12 (6.4*e* − 01)	10 (1.6*e* − 01)	7 (7.7*e* − 01)
$\frac{1}{64}$	22 (1.6*e* − 01)	16 (5.14*e* − 01)	11 (1.6)	7 (5.3*e* − 01)
$\frac{1}{128}$	22 (1.64)	16 (2.58)	11 (2.17)	7 (4.45)

**Table 3:** Number of iterations and CPU time for Braess-Sarazin (2diag(*A*), *v*(1,1)) multigrid *W*-cycle at different levels of refinement, tolerance = 10^−6^.

Levels	MG-*W*-cycle			
	*v*(1,1)	*v*(2,2)	*v*(3,3)	*v*(4,4)
	Iter(cpu time (sec))	Iter(cpu time (sec))	Iter(cpu time (sec))	Iter(cpu time (sec))
$\frac{1}{16}$	16 (8.3*e* − 02)	11 (8.4*e* − 02)	7 (3.5*e* − 02)	5 (9*e* − 02)
$\frac{1}{32}$	17 (6*e* − 01)	12 (6*e* − 01)	7 (1.2*e* − 01)	5 (7.1*e* − 01)
$\frac{1}{64}$	17 (5*e* − 01)	13 (5.1*e* − 01)	8 (1.89)	6 (5.6*e* − 01)
$\frac{1}{128}$	16 (1.98)	13 (3.24)	8 (2.13)	6 (5.78)

**Table 4:** Number of iterations and CPU time for multigrid (*V*-cycle) with different smoothers and smoother preconditioner approximations at different levels of refinement, tolerance = 10^−6^.

Levels	MG-*V*-cycle(3,3)			
	DGS	IUzawa	Braess-Sarazin	PMINRES
	Iter(cpu time (sec))	Iter(cpu time (sec))	Iter(cpu time (sec))	Iter(cpu time (sec))
$\frac{1}{16}$	18 (8.6*e* − 02)	13 (6.3*e* − 02)	9 (2.1*e* − 02)	14 (7.1*e* − 02)
$\frac{1}{32}$	19 (7.4*e* − 01)	14 (2.0*e* − 01)	10 (1.6*e* − 01)	16 (6.5*e* − 01)
$\frac{1}{64}$	19 (3.12)	14 (1.64)	11 (1.6)	16 (4.898)
$\frac{1}{128}$	19 (8.66)	14 (3.2)	11 (2.17)	16 (7.97)

**Table 5:** Number of iterations and CPU time for multigrid (*W*-cycle) with different smoothers and smoother preconditioner approximations at different levels of refinement, tolerance = 10^−6^.

Levels	MG-*W*-cycle(3,3)			
	DGS	IUzawa	Braess-Sarazin	PMINRES
	Iter(cpu time (sec))	Iter(cpu time (sec))	Iter(cpu time (sec))	Iter(cpu time (sec))
$\frac{1}{16}$	15 (9.8*e* − 02)	9 (7.1*e* − 02)	7 (3.5*e* − 02)	15 (8.5*e* − 02)
$\frac{1}{32}$	16 (8.3*e* − 01)	10 (3.4*e* − 01)	8 (1.2*e* − 01)	14 (5.1*e* − 01)
$\frac{1}{64}$	17 (5.76)	9 (2.56)	8 (1.89)	14 (6.43)
$\frac{1}{128}$	17 (9.87)	9 (4.32)	8 (2.13)	14 (8.55)

**Table 6:** Number of iterations and CPU time of iterations for multigrid (*V*-cycle) with different smoothers and using one *v*-cycle multigrid preconditioner approximation (for both $\hat{A}$ and $\hat{S}$) at different levels of refinement, tolerance = 10^−6^.

Levels	MG-*V*-cycle(3,3)			
	DGS	IUzawa	Braess-Sarazin	PMINRES
	Iter(cpu time (sec))	Iter(cpu time (sec))	Iter(cpu time (sec))	Iter(cpu time (sec))
$\frac{1}{16}$	14 (4*e* − 01)	10 (3.4*e* − 02)	6 (1.3*e* − 02)	13 (4.4*e* − 02)
$\frac{1}{32}$	15 (2.1*e* − 01)	10 (2.3*e* − 01)	7 (1.5*e* − 01)	14 (5.4*e* − 01)
$\frac{1}{64}$	16 (2.11)	11 (2.01)	8 (1.01)	14 ()
$\frac{1}{128}$	16 (4.56)	11 (3.67)	8 (2.02)	14 (9.01)

**Table 7:** Number of iterations and CPU time for multigrid (*W*-cycle) with different smoothers and using one *v*-cycle multigrid preconditioner approximation (for both $\hat{A}$ and $\hat{S}$) at different levels of refinement, tolerance = 10^−6^.

Levels	MG-*W*-cycle(3,3)			
	DGS	IUzawa	Braess-Sarazin	PMINRES
	Iter(cpu time (sec))	Iter(cpu time (sec))	Iter(cpu time (sec))	Iter(cpu time (sec))
$\frac{1}{16}$	11 (6.7*e* − 01)	8 (5.6*e* − 02)	5 (3.2*e* − 02)	13 (5.8*e* − 02)
$\frac{1}{32}$	12 (2.4*e* − 01)	9 (3.2*e* − 01)	6 (2.2*e* − 01)	14 (7.3 − 01)
$\frac{1}{64}$	13 (4.62)	9 (4.31)	6 (1.01)	15 (3.21)
$\frac{1}{128}$	13 (7.009)	9 (3.35)	6 (2.98)	15 (6.23)

**Table 8:** Changes in the ‖∇·**u**‖_Ω_­ estimated velocity divergence error using multigrid *V*-cycle. η: the global error estimator using different smoothers from one level to the other.

Levels	‖∇·**u**‖_Ω_		η	
	IUzawa	Braess-Sarazin	IUzawa	Braess-Sarazin
$\frac{1}{16}$	3.2*e* − 001	2.7*e* − 002	7.97*e* − 002	1.81*e* − 001
$\frac{1}{32}$	1.24*e* − 002	1.2*e* − 002	3.08*e* − 002	1.44*e* − 001
$\frac{1}{64}$	5.7*e* − 003	5.93*e* − 003	1.15*e* − 003	1.12*e* − 002
$\frac{1}{128}$	3.18*e* − 003	3.93*e* − 003	4.8*e* − 003	1.12*e* − 002
